# East African cassava mosaic-like viruses from Africa to Indian ocean islands: molecular diversity, evolutionary history and geographical dissemination of a bipartite begomovirus

**DOI:** 10.1186/1471-2148-12-228

**Published:** 2012-11-27

**Authors:** Alexandre De Bruyn, Julie Villemot, Pierre Lefeuvre, Emilie Villar, Murielle Hoareau, Mireille Harimalala, Anli L Abdoul-Karime, Chadhouliati Abdou-Chakour, Bernard Reynaud, Gordon W Harkins, Arvind Varsani, Darren P Martin, Jean-Michel Lett

**Affiliations:** 1 CIRAD, UMR PVBMT, Pôle de Protection des Plantes, 7 Chemin de l’IRAT, Saint-Pierre, Ile de La Réunion, 97410, France; 2 Université de La Réunion, UMR PVBMT, Pôle de Protection des Plantes, 7 Chemin de l’IRAT, Saint-Pierre, Ile de La Réunion, 97410, France; 3 Service de Protection des Végétaux - Direction de l’Agriculture et de la Forêt, B.P 103, Mamoudzou, Mayotte, 97600, France; 4 Institut National de Recherche pour l’Agriculture, la Pêche et l’Environnement, BP289, Moroni, Grande Comore, Union des Comores; 5 South African National Bioinformatics Institute, University of the Western Cape, Cape Town, South Africa; 6 School of Biological Sciences, University of Canterbury, Private Bag 4800, Christchurch, New Zealand; 7 Biomolecular interaction centre, University of Canterbury, Private Bag 4800, Christchurch, New Zealand; 8 Electron Microscope Unit, University of Cape Town, Rondebosch, 7701, Cape Town, South Africa; 9 Institute of Infectious Diseases and Molecular Medicine, University of Cape Town, Observatory 7925, Cape Town, South Africa

**Keywords:** Cassava, Begomoviruses, Dissemination, Africa, Phylogeography, Recombination

## Abstract

**Background:**

Cassava (Manihot esculenta) is a major food source for over 200 million sub-Saharan Africans. Unfortunately, its cultivation is severely hampered by cassava mosaic disease (CMD). Caused by a complex of bipartite cassava mosaic geminiviruses (CMG) species (Family: *Geminivirideae*; Genus: *Begomovirus*) CMD has been widely described throughout Africa and it is apparent that CMG's are expanding their geographical distribution. Determining where and when CMG movements have occurred could help curtail its spread and reveal the ecological and anthropic factors associated with similar viral invasions. We applied Bayesian phylogeographic inference and recombination analyses to available and newly described CMG sequences to reconstruct a plausible history of CMG diversification and migration between Africa and South West Indian Ocean (SWIO) islands.

**Results:**

The isolation and analysis of 114 DNA-A and 41 DNA-B sequences demonstrated the presence of three CMG species circulating in the Comoros and Seychelles archipelagos (East African cassava mosaic virus, EACMV; East African cassava mosaic Kenya virus, EACMKV; and East African cassava mosaic Cameroon virus, EACMCV). Phylogeographic analyses suggest that CMG’s presence on these SWIO islands is probably the result of at least four independent introduction events from mainland Africa occurring between 1988 and 2009. Amongst the islands of the Comoros archipelago, two major migration pathways were inferred: One from Grande Comore to Mohéli and the second from Mayotte to Anjouan. While only two recombination events characteristic of SWIO islands isolates were identified, numerous re-assortments events were detected between EACMV and EACMKV, which seem to almost freely interchange their genome components.

**Conclusions:**

Rapid and extensive virus spread within the SWIO islands was demonstrated for three CMG complex species. Strong evolutionary or ecological interaction between CMG species may explain both their propensity to exchange components and the absence of recombination with non-CMG begomoviruses. Our results suggest an important role of anthropic factors in CMGs spread as the principal axes of viral migration correspond with major routes of human movement and commercial trade. Finer-scale temporal analyses of CMGs to precisely scale the relative contributions of human and insect transmission to their movement dynamics will require further extensive sampling in the SWIO region.

## Background

Cassava (*Manihot esculenta* Crantz) is cultivated as a subsistence crop in developing countries across the world where its roots represent an indispensable source of dietary carbohydrates for over 700 million people [1]. In sub-Saharan Africa and the South West Indian Ocean islands (SWIO), cassava has become the major staple food crop constituting the main part of the diet of traditional households (ranked first in crop production; FAOSTAT 2010).

Because cassava produces acceptable yields even on very marginal soils and under drought conditions, and because it is a perennial that has no set maturation point and can be harvested any time, it is often cultivated as a famine reserve in developing countries.

Unfortunately, cassava cultivation is associated with a wide range of diseases that seriously undermine the food and economic security in these countries, the most notable of which is cassava mosaic disease (CMD), caused by a complex of cassava mosaic geminiviruses (CMG’s, Family *Geminiviridae*, Genus *Begomovirus*) [1]. CMD is currently the most damaging plant virus disease in the world with an epidemic in East and Central Africa, causing annual crop losses valued at between US$1.9–2.7 billion and a famine that has likely caused the deaths of thousands of people [1].

CMG’s possess bipartite genomes, with genome components, called DNA-A and DNA-B, comprising 2.7 kb circular single-stranded DNA molecule. Both components are necessary for successful infection of cassava. While DNA-A encodes proteins and regulatory elements responsible for replication, encapsidation functions and control of gene expression, DNA-B encodes proteins enabling viral movement [2]. The cognate components of a particular virus share an approximately 200 nucleotide long homologous sequence, called the common region (CR), which is involved in the initiation of replication by the DNA-A encoded replication associated protein (Rep) and that generally exhibits more than 85% sequence identity between the components.

Interestingly, whereas cassava originates from South America [3], the African CMG’s are endemic to Africa and are likely recent descendants of geminiviruses adapted to infect indigenous uncultivated African plant species [4]. Therefore the adaptation of CMG’s to cassava could have only commenced, either after cassava was introduced to West Africa in the Gulf of Guinea during the 16^th^ century, or after it was introduced to East Africa and the SWIO islands in the 18^th^ century [4,5].

Since the initial characterization in the early 1980s of the “first” CMG species, *African cassava mosaic virus* (ACMV) [6], it has subsequently been discovered that African CMG’s in fact consist of at least six distinct begomovirus species including *South African cassava mosaic virus* (SACMV), *East African cassava mosaic virus* (EACMV), *East African cassava mosaic Cameroon virus* (EACMCV), *East African cassava mosaic Zanzibar virus* (EACMZV), *East African cassava mosaic Malawi virus* (EACMMV), and *East African cassava mosaic Kenya virus* (EACMKV) [7,8]. Recently, two putative species have also been newly described: *African cassava mosaic Burkina Faso virus* (ACMBFV, [9]) and *Cassava mosaic Madagascar virus* (CMMGV, [10]).

Besides being transmitted by the whitefly *Bemisia tabaci*, for which numerous invasive species, formerly referred to as biotypes [11], have been described, transmission via human mediated transport of infected tubers is today postulated to play an important role in the spread of CMG’s. Currently, various CMG species are widely distributed throughout Africa [1,12] and ongoing monitoring of cassava indicates that some CMG species are in the process of expanding their geographical ranges.

Whereas several studies have indicated that single stranded DNA viruses in general, and begomoviruses in particular, have surprisingly high substitution rates [13-18], begomoviruses also have a great propensity to recombine [19-21]. The co-existence of different CMG species in one geographical area can result in the frequent co-occurrence within individual cells of infected cassava plants (or any other suitable host species for that matter) of genetically distinct CMG species. A natural consequence of the recombination dependent mechanism via which begomoviruses replicate [2] is the occurrence within such co-infections of numerous inter-species recombinant genomes. Although the vast majority of such recombinants most likely never survive for long enough to be detected, the occasional generation, emergence and proliferation of a new, and presumably highly-fit, recombinant variant from such mixed infections can have extremely long-term negative consequences for cassava farmers.

Probably the cardinal example of this phenomenon is the emergence of the Uganda strain of EACMV (also called the “Uganda variant” or EACMV-UG). At some time before 1990, a recombination event resulted in a large portion of the CP gene of an EACMV genome being replaced by a homologous CP gene fragment of ACMV. First described in northern Uganda in the early 1990s, it was soon directly and quite convincingly implicated as one of the primary causes of a new extremely virulent and rapidly spreading form of CMD [22,23]. Although the precise biological effect of the recombination event that generated EACMV-UG remains unknown, it is nevertheless abundantly clear that the diversification of begomoviruses in general, and CMG’s in particular, is being largely fuelled by the relentless ongoing mixing of genetic lineages that occurs within mixed infections.

Besides homologous recombination, another form of genetic recombination that is known to occur during the evolution of viruses that have multiple genome components (such as CMG’s) is the re-assortment or swapping of components between different virus species (also known as pseudo-recombination). As is the case with homologous recombinants, all possible genome component re-assortments are not equally viable. The most viable begomovirus re-assortants tend to be those between closely related species [24] implying that co-evolved genetic interactions between the DNA-A and DNA-B components of bipartite begomoviruses are crucial for their successful cooperation during infections. Prime amongst these interactions is the need for the DNA-A expressed replication associated protein to recognise and nick the rolling circle replication origin within the DNA-B CR. Crucially, homologous recombination between the DNA-A and DNA-B common regions is likely an important mechanism whereby DNA-A components maintain replicational control over their cognate DNA-B components. Indeed, it has been experimentally demonstrated that mutated ACMV-B components can have their CR sequences restored by homologous recombination with closely related DNA-A CR sequences [25]. In fact, such assertion of replicational control by begomoviral DNA-A components over other cognate components has even been observed in satellite molecules which, despite not normally having a CR, can occasionally acquire one through non-homologous recombination with a DNA-A [26,27].

As the complexity of the CMD pandemic becomes increasingly apparent, it is worthwhile, both for purely historical reasons and from a scientific-epidemiological perspective, to understand the key factors associated, firstly, with the successful emergence of the various CMG species, and, secondly, with their constitution of the CMD complex. Given the sampling locations, sampling dates and full genome sequences of enough CMG isolates it should be possible to query the multiple genetic imprints left in the course of their emergence as cassava pathogens to statistically retrace the historical migration rates and dissemination routes of these viruses across sub-Saharan Africa and the SWIO islands.

Here we analyse hundreds of CMG isolates, including many sequences isolated for the first time from the SWIO Seychelles and Comoros archipelagos, in an attempt to understand the relative contributions of mutation, recombination, genome component re-assortment and migration to the evolutionary dynamics of these viruses. We use precise sampling coordinates, full CMG genome sequences, and detailed homologous recombination and genome component re-assortment analyses to map the distribution of CMG’s in Africa, Comoros and Seychelles sampled during the period 1996–2009. We have employed Bayesian phylogeographic techniques to retrace, prior to these sampling dates, the spatial dynamics of EACMV-like viruses within Africa and between the SWIO Islands to demonstrate frequent, likely ongoing, and possibly human mediated, long range movements of CMG’s across these regions.

## Results and discussion

### Three coexisting EACMV-like virus species

A total of 114 full DNA-A and 41 full DNA-B components were cloned and sequenced from dried cassava leaf samples collected in Grande Comore (GC, 42 DNA-A and 6 DNA-B), Anjouan (AJ, 12 DNA-A and 9 DNA-B), Mohéli (MO, 17 DNA-A and 4 DNA-B), Mayotte (YT, 32 DNA-A and 20 DNA-B) and the Seychelles archipelago (SC, 11 DNA-A and 2 DNA-B). In addition to these full genome sequences, 43 partial DNA-A sequences of the core capsid protein (CP) gene were obtained (see Additional file 1: Table S1 for details).

**Figure 1:**
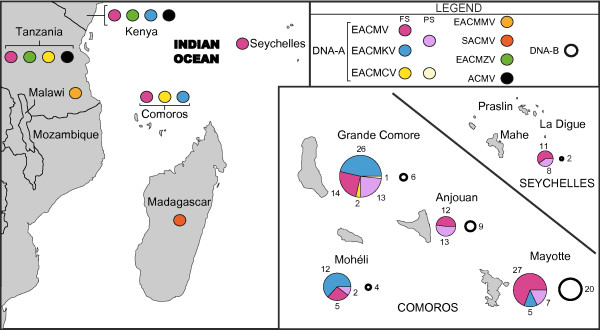
**Repartition map of the seven African CMG’s in East Africa, the Comoros archipelago and the Seychelles archipelago.** The map on the left describes the general repartition of the seven DNA-A species in the area. The map at the bottom right zooms in on the archipelagos with the number and composition of samples from each island represented with pie charts. The colour code is available on the top right of the figure with FS indicating full genome sequences and PS indicating partial genome sequences.

Out of the eight species of CMG’s characterised in Africa, only complete sequences of EACMV (n = 69), EACMKV (n = 43) and EACMCV (n = 2) were identified amongst the Comoros and the Seychelles archipelago samples. The partial sequences of the core CP genes of 43 isolates were also all classifiable as belonging to one of these three species (EACMV/EACMKV, n = 42; EACMCV, n = 1), although it was not possible to differentiate between EACMV and EACMKV isolates based on the CP alone. Whereas EACMV was identified on every island sampled, EACMKV was found everywhere other than in the Seychelles archipelago and in Anjouan. EACMCV was only detected in Grande Comore (Figure 1). Interestingly, we noticed large variability in the CMG species composition among islands (Figure 1). While in Mayotte and Anjouan EACMV represented the vast majority of the isolates (Mayotte: 84%; Anjouan: 100%), on Grande Comore and Mohéli it represented only approximately a quarter of CMG isolates, the remainder being mostly EACMKV (Grande Comore: 62%; Mohéli: 71%).

Importantly, when examining the phylogenetic tree of the DNA-A sequences (Additional file 2: Figure S1), several distinct variants of EACMV and EACMKV are specific to the SWIO. All of these (1) share higher sequence identity to CMG isolates from mainland Africa than to the other SWIO variants and (2) display complex geographical distributions. Together with the variation in CMG species compositions from one island to the next, this suggests a complex history of virus migrations throughout the SWIO region.

Regarding the DNA-B components, 17 sequences were obtained from samples infected with EACMV DNA-A sequences and seven from samples infected with EACMKV DNA-A sequences. The remaining 17 DNA-B sequences were isolated from samples from which no DNA-A was obtained. Based on BLAST searches, the majority of these sequences appeared most closely related to EACMKV DNA-B components (n = 34) with seven sequences being most closely related to EACMV DNA-B sequences. However, it is important to note that, as has previously been indicated [28], there is no clear demarcation between EACMV, EACMKV, EACMZV and SACMV DNA-B sequences, and that species assignment based on DNA-B sequences alone remains difficult for CMG’s. Interestingly, the two DNA-B sequences isolated from the Seychelles samples present lower sequence identity (less than 88%) to EACMV-like DNA-B components than did the rest of the CMG DNA-B’s analysed.

The difficulties inherent in classifying DNA-B sequences were confirmed by their phylogenetic analysis (Additional file 3: Figure S2). The DNA-B sequences of EACMV, EACMKV, EACMZV and SACMV do not cluster according to the species classification of their associated DNA-A sequences and the two DNA-B sequences from the Seychelles clustered within a separate clade distinct from the other SWIO island CMG DNA-B’s sequences that was most closely related to EACMCV (Additional file 3: Figure S2). In this phylogenetic tree, no clear clustering of sequences based on sampling location was apparent, again suggesting both multiple-introductions of these DNA-B lineages to individual islands and complex historical migration patterns between the SWIO islands and mainland Africa.

### Recombination analyses

As recombination is a major process influencing the evolution of single stranded DNA viruses in general and begomoviruses in particular, we searched for evidence of (1) CMG sequence fragments being transferred into the genomic backgrounds of other species (i.e. events with CMG donors) and (2) genomic fragments of other species being transferred into mostly CMG-like genomic backgrounds (i.e. events with CMG recipients).

To do so, we constituted a dataset comprising the 114 full-length DNA-A sequences from this study with 264 DNA-A and DNA-A-like sequences representing all available CMG sequences from Africa and Asia including both monopartite and bipartite sequences of African begomoviruses, and additional sequences of legumoviruses, curtoviruses and topocuviruses available in GenBank.

Of the 15 detected recombination events involving CMG DNA-A sequences (Table 1), eight involve obvious inter-species recombination, while the others lack clear identification of at least one parental sequence but were nevertheless also likely inter-species recombinants. Intra-species recombination was not detected in our analyses, probably due to the difficulties associated with detecting recombination between very closely related sequences within such large datasets [29]. Only two of the inter-species recombination events detected involve exchanges between two EACMV-like species, while other events represent exchanges between an EACMV-like and ACMV (3 events) or tomato-infecting begomoviruses (3 events).

**Table 1:** List of recombination events inferred in EACMV-like sequences

	**Event number**	Recombinant *	**Region**		Minor Parent *	Major Parent *	**Methods**^**α**^	**P-value**^**β**^
			**Begin**	**End**				
DNA-A	1	EACMV-UG	545	1008	ACMV	EACMV	RG**B**MCST	1.2x10^-71^
	2	EACMCV	1052	1872	Unknown	EACMV	RGBMCS**T**	1.3x10^-89^
	3	EACMKV, EACMZV, SACMV	1835	9	ToLCV-like	EACMV	RG**B**MCST	6.7x10^-53^
	4	EACMV-KE	1878 ^$^	2083	EACMZV	EACMV	RGBMCS**T**	1.4x10^-24^
	5	EACMMV, SACMV	178	999 ^$^	ToLCV/TYLCV-like	EACMV	RGBMCS**T**	4.9x10^-20^
	6	EACMMV	1843 ^$^	2311	EACMKV	EACMV	RG**B**MCST	6.0x10^-15^
	7	EACMZV	1840 ^$^	2184	EACMKV	Unknown	RGBMC**S**T	6.7x10^-22^
	8	EACMV, EACMMV	2052 ^$^	2360	Unknown	Unknown	RGBMC**S**T	7.2x10^-16^
	9	EACMV	1821	1935	ACMV	EACMV	**R**GBs	1.0x10^-10^
	10	EACMKV, SACMV	2625	2742 ^$^	EACMV-like	Unknown	RGBMC**S**	1.3x10^-11^
	11	ACMV	1788	1856	EACMV	ACMV	**R**gB	3.3x10^-5^
	12	ToLCCV	2591	2646	EACMV	ToLCCV	Rg**B**S	9.1x10^-5^
	13	EACMV-like	190	451	Unknown	Unknown	rBc**S**	5.7x10^-12^
	14	EACMCV	1056 ^$^	1126	EACMCV	Unknown	rgB**S**	6.0x10^-5^
	15	EACMV	2662	2684	Unknown	EACMV	**R**gb	3.7x10^-2^
DNA-B	1	CMMGV	1564	2688	Unknown	EAC	RGBMCS**T**	1.6x10^-78^
	2	EACMV-[SC]	1124	1454	EACMCV-like	EAC	**R**GBMCST	5.8x10^-21^
	3	EAC	2461	2704	EAC	EAC	**R**GBMCST	3.3x10^-12^
	4	EAC	2672	5	Unknown	EAC	RG**B**MCST	5.6x10^-13^
	5	CAM	1496	2503	EACMV-[SC]-like	Unknown	RGB**M**C	2.2x10^-9^
	6	EAC	2612	37	EAC	EAC	RGBMC**S**	1.3x10^-11^
	7	EAC	422	1647	Unknown	EAC	RMC**S**T	1.3x10^-6^
	8	EAC	723	1143	Unknown	EAC	**R**GBMCSt	3.2x10^-6^
	9	EAC	1718	2751	EAC	EAC	BMC**S**T	1.7x10^-15^

^*^ EAC corresponds to B components associated with EACMV, EACMKV, EAMCZV; CAM corresponds to EACMCV-B; CMMGV, Cassava mosaic Madagascar virus[MG:Tol:06]; EACMV-[SC] corresponds to East African cassava mosaic virus isolated in Seychelles; ToLCV, Tomato leaf curl virus; TYLCV, Tomato yellow leaf curl virus; ToLCCV: Tomato leaf curl Comoros virus.

^$^ Breakpoints not inferred by RDP.

^α^ R: RDP; G: GENECONV; B: Bootscan; M: MaxChi; C: Chimaera; S: SiScan; T: 3Seq. Upper-case letters imply that a method detected recombination with a multiple comparison corrected P-value < 0.05, lower-case letters imply that the method detected recombination with a multiple comparison corrected P-value > 0.05

^β^ The reported P-value is for the method in bold type and is the best P-value calculated for the region in question.

Previous studies have demonstrated that recombination is a particularly important process in the diversification of EACMV-like CMG species that has resulted in the emergence of new epidemiologically-important CMG variants (e.g. the EACMV-Uganda severe variant; [23]), or species (e.g. the EACMMV, [30]; for review [12]). Despite our analyses confirming both the presence of multiple CMG species on various SWIO islands and the pervasiveness of inter-species recombination amongst CMG’s, we were unable to identify any DNA-A recombination event that was unique to SWIO CMG lineages. This may suggest that, despite the presently overlapping geographical ranges of different CMG species on the SWIO islands, mixed infections of these viruses and recombination events between them on these islands have not presently yielded any major epidemiologically relevant lineage. It is plausible that the introduction of these viruses to the SWIO islands may have simply been too recent either for such recombinants to have emerged yet or for them to have proliferated to the point where they would be detectable in a survey such as that described here.

The general recombination profile revealed by our analysis (Additional file 4: Figure S3) is characterised by the absence of significant breakpoint hot spots in the inter-genic region (IR), contrasting with profiles of monopartite begomoviruses obtained in previous studies [20]. The small number of events identified in our dataset, combined with the inability to clearly locate many breakpoints must most likely explain these results. However, recombination hot-spots are identified around nucleotide positions 1000 (4 of 15 events with breakpoints between positions 950 and 1100) and 1800 (7 of 15 events with breakpoints between positions 1750 and 1900) which correspond respectively to previously identified recombination hotspots near the end of ORF AV1 (encoding the capsid protein – CP), and the central region of ORF AC1 (encoding the Replication-associated protein – Rep) (Table 1).

A DNA-B dataset of 168 begomoviruses, containing our 41 new sequences, along with representatives of the other diverse cassava-infecting begomovirus DNA-B sequences, was analysed in the same manner as described for the DNA-A dataset. Nine recombination events were identified in the dataset, with two of those being unique to SWIO sequences (Table 1). A recombination event (event 2 of DNA-B, Table 1) was detected in the two Seychelles DNA-B sequences, spanning the IR between ORFs BV1 and BC1 (position 1124 to 1454 relative to EACMV-[AJ:Oua:AJ03AN3:2004], accession JF909200). Whereas the genome region between the detected breakpoints was distantly related to an EACMCV DNA-B sequence (83% identity with EACMCV-[TZ1], accession AY795989), the remainder of the genomes of these Seychelles DNA-B sequences resemble those of EACMV-like group DNA-B sequences. This recombination event most likely explains why the two Seychelles DNA-B sequences appear as outliers within the CMG DNA-B sequences phylogenetic tree.

Recombination event 8 listed in Table 1 was detected only within the DNA-B sequence of an isolate of EACMV from Anjouan (EACMV-[AJ:Bam:AJ29AQ1:2009]; JF909211). The DNA-B of this virus was closely related to EACMV DNA-B sequences (97,2% identical to EACMV-[AJ:Dzi:AJ10AK1:2005]; accession JF909202) except in the 3' half of the BV1 ORF (encoding the nuclear shuttle protein – NSP, position 723 to 1143) which was apparently derived from a virus species/isolate that is currently unsampled. Due to this recombination event, the BV1 ORF of EACMV-[AJ:Bam:AJ29AQ1:2009] (JF909211) seems non-functional (it has a premature stop codon), and this recombinant sequence potentially represents a non-viable variant.

The remaining events involve DNA-B sequences belonging to the clade EACMV/EACMKV/EACMZV/SACMV, for which, as mentioned previously, species demarcation is difficult. Importantly, however, none of these events were unique to the SWIO viruses.

Two recombination hotspots, mapping with the IR region (2500–50) and the core of the BC1 ORF coding for the MP protein (~1700pb) were detected and partially confirms results obtained in previous studies (Additional file 4: Figure S3).

### CMG diversity on the SWIO islands is fuelled by multiple introductions from East Africa

Clearly apparent from the phylogenetic reconstruction of CMG DNA-A sequences (Additional file 2: Figure S1), the history of CMG migrations onto the SWIO islands is complex and most likely involves multiple introduction events including migrations from East Africa and between islands. To precisely reconstruct the pathways and evolutionary time-frame associated with CMG diversification and movements, we employed the probabilistic framework implemented in the computer program BEAST [31]. Given sampling locations and dates for a set of sequences, BEAST permits the spatial-temporal reconstruction of plausible movement pathways underlying the observed geographical distributions of the analysed sequence sample. On the basis of GPS sampling coordinates, we defined seven distinct geographical groups corresponding to West and East/Centre Africa (Centre and East Africa for full-genome DNA-B – FG-B – dataset), the Seychelles, and each of the four Comoros islands separately (Additional file 5: Figure S4). We then used the discrete phylogeographic model [32] to reconstruct migration routes of CMG’s between these localities.

As recombination is known to confound molecular clock analyses, in addition to the full-genome DNA-A (FG-A) dataset, we constituted a mostly recombination-free (details in [33]) core CP ORF (CP) dataset. Consistent with previous work on large begomovirus datasets that indicated that the core CP region is a recombination cold-spot [16], there was an absence of detectable recombination breakpoints within this region of the CMG’s and their closest relatives. The final dataset that we constructed for these analyses was a DNA-B dataset (called FG-B).

For each of these datasets, all the new sequences described in this study were aligned with all currently available EACMV-like sequences from GenBank with an associated sampling date and location. This yielded 226, 213 and 92 sequences for the FG-A, CP and FG-B datasets respectively (see Additional file 1: Table S1 for details).

We analysed each of these datasets using BEAST to infer the time when and the place where the most recent common ancestor of the EACMV-like viruses originated. While the FG-A analysis indicated that the mean substitution rates during EACMV-like virus evolution has been approximately 1.27 x 10^-3^ subs/site/year (95% highest posterior density - HPD - interval ranging from 9.08 x 10^-4^ to 1.64 x 10^-3^), the CP analysis indicated a rate of 1.93 x 10^-3^ subs/site/year (95% HPD ranging from 1.26 x 10^-3^ to 2.64 x 10^-3^). These substitution rate estimates are consistent with previously published estimates of substitution rates for these viruses [14]. For the DNA-B, however, our estimate of 2.35 x 10^-3^ subs/site/year (95% HPD ranging from 1.36 x 10^-3^ to 3.35 x 10^-3^) was faster than both those estimated for the DNA-A datasets, and that estimated for EACMV DNA-B in a previous study (1.33 x 10^-4^; HPD between 1.06 x 10^-5^ and 3.39 x 10^-4^; [14]).

It is important to point out here that, due to the relatively short time span over which the analysed samples were collected (1996 to 2009 but with 95% of the samples obtained between 2000 and 2009), the substitution rates inferred are probably more reflective of short-term mutation rates and not the longer-term substitution rates of EACMV-like viruses [15]. This is because it is expected that over the 13 year sampling period there would have likely been insufficient time for purifying selection to remove many slightly deleterious mutations that would ultimately be purged from EACMV populations over longer time-frames (which if purged would result in lower substitution-rate estimates). For this reason, it is probable that our datasets will be ineffective for accurately estimating the dates of the deeper nodes of the EACMV-like virus phylogenetic tree.

**Figure 2:**
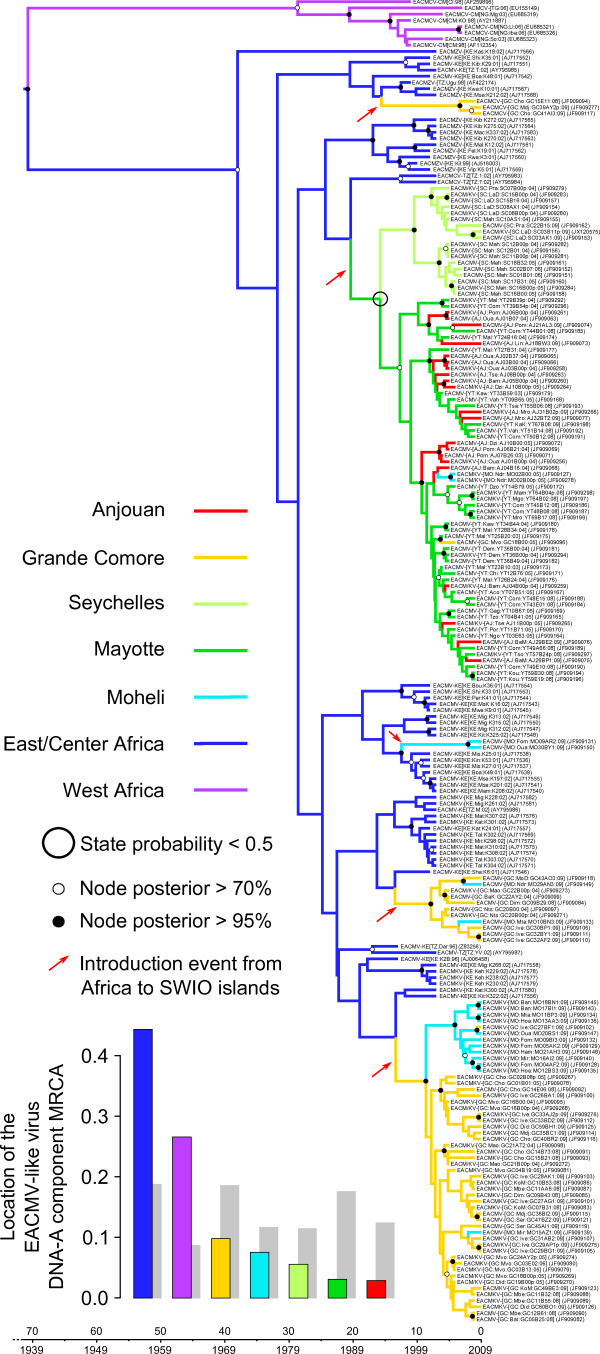
**Maximum clade credibility trees constructed from the EACMV-like capsid protein (CP) dataset.** Branches are coloured according to the most probable location state of the node on their right (i.e. the likely geographical location of the ancestral sequence represented by this node). The large black circle around one of the nodes indicates that the state probability at this node is less than 0.5 (i.e. there is less than 50% confidence in the indicated location being the actual place where this ancestral sequence existed). The time-scale of evolutionary changes represented in the tree is indicated by the scale bar below it. Whereas filled circles associated with nodes indicate > 95% posterior probability support for the branches to their left, open circles indicate nodes with > 70% posterior support for these branches. Nodes to the right of branches with < 70% support are left unlabelled. The bar graph on the left corner indicates location probabilities of the node at the root of the tree (which is the node representing the last common ancestor of all the sequences represented in the tree). Grey bars represent the probabilities obtained with randomisation of the tip locations. Probable introduction events from Africa to the SWIO islands are indicated with red arrows.

Whereas the most recent common ancestor (MRCA) of the EACMV-like viruses was estimated to be 1880 (95% HPD: 1786–1945) using the FG-A dataset (Additional file 6: Figure S5) it was dated to only 1938 (95% HPD: 1867–1982) using the CP dataset (Figure 2). Using the FG-B dataset, the MRCA was dated to 1921, a date that is included in all the other estimated HPDs (HPD: 1820–1980). These contradictory date estimates highlight another potential bias introduced by recombination to the full genome datasets. It is expected that with the FG-A dataset, the much older dates of the last common ancestors of the divergent recombinationally acquired “non-EACMV-like” genome fragments would have pushed the estimated date of the EACMV-like virus MRCA much deeper into the past [13,34] (i.e. the estimated date is expected to be somewhere between the date of the MRCA of the recombinationally acquired genomic tracks and the date of the MRCA of the rest of the genome).

Therefore, when not stated otherwise, below we present dates of ancestral sequences using the core CP dataset. Importantly, though, since these dates will still be strongly upwardly biased due to the analysed sequences being sampled over such a short time-period, we will instead primarily focus on phylogeographic inferences since these will not have been influenced by this unavoidable bias. Moreover, while we use both FG-A and CP datasets analyses to describe phylogeographic patterns for CMG's in these islands, more credence should be given to inferences supported by the recombination-free CP dataset, as recombination can also have confounding effects on FG-A dataset phylogeographic reconstruction.

**Figure 3:**
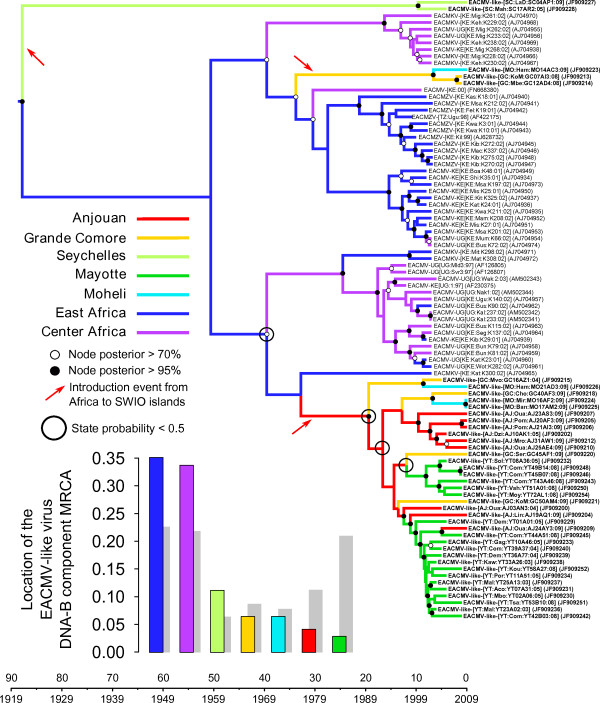
**Maximum clade credibility trees constructed from the EACMV-like DNA-B Full genome (FG-B) dataset.** Branches are coloured according to the most probable location state of their descendant nodes, with black circled nodes indicating nodes with state probabilities < 0.5. The time-scale of evolutionary changes represented in the tree is indicated by the scale bar below it. Whereas filled circles associated with nodes indicate > 95% posterior probability support for the branches to their left, open circles indicate nodes with > 70% posterior support for these branches. Nodes to the right of branches with < 70% support are left unlabelled. The bar graph on the left corner indicates the location probabilities of the node at the root of the tree that represents the most recent common ancestor of all the sequences represented in the tree. Grey bars represent the probabilities obtained with randomisation of the tip locations. Probable introduction events from Africa to the SWIO islands are indicated with red arrows.

Not surprisingly, both the FG-A and CP analyses clearly indicated that the MRCA of the EACMV-like viruses probably resided on the African mainland (posterior state probability or PSP = 0.96 and 0.71 for FG-A and CP datasets respectively; see Additional file 6: Figure S5 and Figure 2). If East/Centre Africa is more strongly supported as the root location than West Africa, it is impossible with our grouping design to provide definitive results at a finer spatial scale. The most probable geographical origin of DNA-B MRCA is also likely mainland Africa (PSP = 69%; Figure 3).

Although our inference of the location of the EACMV-like virus MRCA would have been uninfluenced by the short time-frame over which samples were collected, it could have potentially been biased by the fact that sample sizes differed among locations. We therefore repeated our analyses using a configuration that randomizes the assigned locations of the sequences along the course of the analyses. If with these settings we again encountered mainland Africa as the likely origin of the EACMV-like viruses, it would imply that this result may have simply been caused by greater numbers of mainland African EACMV sequences having been included in our analysis. The grey “shadows” within the bar graphs in Figure 2, Figure 3 and Additional file 6: Figure S5 indicate the estimates obtained from these location randomized analyses. Importantly, none of these analyses yielded probability estimates for the location of the EACMV-like virus MRCA that were anywhere near as high as those obtained with the real datasets, confirming that our estimation of the EACMV-like virus MRCA’s location was robust to possible sampling biases.

The phylogeographic analyses of the FG-A and CP datasets implied that the current geographical distribution of EACMV-like virus genetic variants could best be explained by the possibility of at least four (for FG-A) or five (for CP) independent introductions of these viruses to the SWIO islands from mainland Africa (see tree branches indicated by red arrows in Figure 2 and Additional file 6: Figure S5). Whereas the four introductions inferred from the analyses of the FG-A dataset all involved movements from Africa to Grande Comore, the CP dataset indicated that movements from Africa had occurred three times to Grande Comore, once to Mohéli and once to Mayotte.

Importantly, the inferred locations of the ancestral sequences at some of the nodes do not have well resolved location estimates (highest location state probability of these estimates was lower than 0.5), and are circled in black in Figure 2 and Additional file 6: Figure S5. Slight changes in these probabilities as might be achieved with a larger sample of sequences from a wider variety of locations, would therefore likely yield different estimates to the number of independent introduction events inferred here. Crucially though, the CP phylogeographic reconstruction is far less ambiguous than that for the FG-A dataset since there is only a relatively high degree of uncertainty regarding the movement of viruses between Africa and the Seychelles: The two almost equally credible scenarios implied by our analysis is that there was a EACMV-like virus which either moved to the Seychelles from Mayotte between 1993 and 1999 (CP dataset HPD: 1985–2002) or was directly introduced to the Seychelles from Africa (depicted in Figure 2 and Additional file 6: Figure S5).

As emphasized before, recombination likely introduces a major bias of the FG-A molecular clock and phylogeographic analyses because of the detrimental effects it has on the accurate inference of phylogenetic trees [35,36]. Here, it is clearly apparent that some of the isolates share the same CP gene but not the remainder of their genomes. The position of some EACMV and EACMKV clades is incongruent between FG-A and CP trees, thus slightly modifying the migration histories inferred using these two datasets.

From the FG-A dataset, the MRCA of all the EACMKV sequences sampled from the Comoros islands infers that this species arrived on Grande Comore in a single founding event that occurred sometime between 1981 and 1988 (FG-A HPD: 1966–1997). This founding lineage likely then moved from Grande Comore to Mohéli and Mayotte between 1997 and 2003 (Additional file 6: Figure S5). Note that over the CP dataset, a major event involves the migration of EACMKV from Africa to Grande Comore between 1995 and 2001 (CP HPD: 1990–2003) but that the isolates from Mayotte and two isolates from Mohéli are regrouped within EACMV clade.

This phenomenon is possibly due to an undetected recombination event transferring the entire CP region between isolates of these species. Such difficult to detect recombination events have been invoked previously as an explanation for the CP genes of isolates belonging to the *Tomato yellow leaf curl virus* (TYLCV) strains IL and Mld being polyphyletic [16].

A single introduction of EACMCV to Grande Comore probably occurred from Africa between 1993 and 2006 (CP dataset HPD: 1987–2008). As only two EACMCV sequences have been isolated on Grande Comore (one in 2008 and one in 2009) no other movement events could be inferred for this species. It is possible that the low prevalence of EACMCV on SWIO islands may be due either to these viruses having only been on the islands for a very short time or because they are in the process of being displaced by another virus.

All the others introductions correspond to isolates of EACMV and likely occurred between 1988 and 2008 (CP dataset HPD 1978–2009).

**Figure 4:**
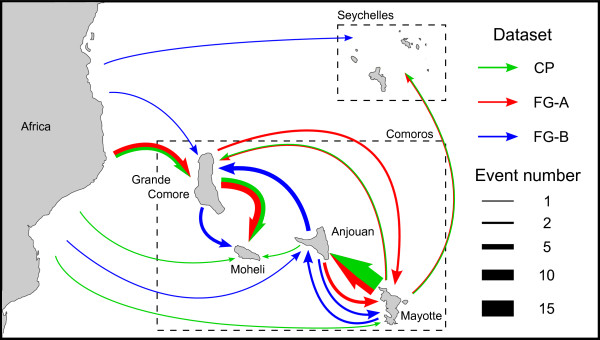
**CMG migrations from East Africa and between SWIO islands.** CMG migration events inferred using the capsid protein (CP, in green); full genome DNA-A (FG-A, in red) and full genome DNA-B (FG-B, in blue) datasets. Arrow colours represent the dataset used to infer each migration event. The thickness of arrows is proportional to the number of independent movements inferred between islands.

Despite the bias exposed earlier, the CP and FG-A datasets both yielded congruent estimates of the migration routes between different islands. Two major migration directions are inferred and strongly supported by Bayes factor (BF) tests. From the FG-A and the CP datasets respectively four and six migrations were inferred from Grande Comore to Mohéli between 1999 and 2009 (CP HPD between 1997 and 2009 with an associated BF ~ 30000). Similarly, between six and fourteen migrations were inferred between Mayotte to Anjouan between 1999 and 2009 (CP HPD between 1997 and 2009 with an associated BF ~ 30000; Figure 4). Although the results very strongly indicate that EACMV have moved frequently and relatively unimpeded between Mayotte and Anjouan (although primarily from Mayotte to Anjouan), it must be stressed in this case that there is a high degree of phylogenetic uncertainty in the inferred locations of the ancestral sequences used to detect some of these individual movements. For example, whereas the CP dataset indicates all movements were from Mayotte to Anjouan, up to three possible movements from Anjouan to Mayotte are indicated for the FG-A dataset. One movement from Mayotte to Grande Comore between 2002 and 2005 (CP dataset 95% HPD between 2001 and 2005) and another from Mohéli to Grande Comore between 2008–2009 (CP dataset 95% HPD between 2007 and 2009) are supported by both datasets, whereas the other migrations are supported by one or the other dataset but not both.

Three migration events from Africa to the SWIO islands could be inferred from the FG-B dataset (Figure 3). The first, at the very base of the MCC tree, implies a possible movement of viruses from Africa to the Seychelles between 1921 and 2000 (HPD: 1819–2004). However, as was indicated earlier, the recombinant nature of these two outlier sequences from the Seychelles may have resulted in their artifactual placement at the root of the MCC tree.

Two other concomitant but phylogenetically distinct EACMV-like virus DNA-B sequence introductions from East Africa to the SWIO are later inferred. One migration occurs from East Africa to Grande Comore between 1975 and 2003 (95% HPD between 1955 and 2007), and then from Grande Comore to Mohéli between 2002 and 2009 (95% HPD: between 1997 and 2009). Unfortunately only three DNA-B sequences belonging to this phylogenetic clade are available and further movements amongst these islands such as those observed with the FG-A and CP datasets were therefore impossible to detect. The second EACMV-like virus DNA-B migration detectable between East Africa and the SWIO islands occurred to Anjouan between 1976 and 1990 (95% HPD between 1955 and 1999). From Anjouan there were likely four migration events to Grand Comore and two to Mayotte all between 1989 and 2009 (95% HPD between 1976 and 2009). Additionally, these analyses inferred two migrations from Grande Comore to Mohéli between 2000 and 2009 (95% HPD between 1995 and 2009), and two from Mayotte to Anjouan between 1997 and 2009 (95% HPD between 1993 and 2009; Figures 3 and 4).

As was found with the EACMV DNA-A migrations, it was difficult to conclusively determine the numbers and directions of DNA-B movements between Mayotte and Anjouan. Importantly, the same two major migration paths between the SWIO islands that were indicated in the FG-A and CP dataset analyses were also identified in the FG-B analysis (Grande Comore/Mohéli, BF for FG-B analysis = 138; Mayotte/Anjouan, BF for FG-B analysis = 57). The DNA-B dataset did, however, also strongly support an additional route (Grande Comore/Anjouan, BF = 4).

In all these analyses, it is important to note that migrations of CMG’s from the SWIO islands to Africa are barely ever detected. A single such event may be apparent in the FG-A phylogeographic reconstruction (see the bi-colour branch on Additional file 6: Figure S5), but this signal could be artifactual since the probabilities of the locations for the supposedly migratory ancestral sequence involved is highly uncertain and could in fact be Africa in general: i.e. the location probabilities for West and East/Centre Africa are respectively 0.11 and 0.27 and sum to 0.39 which is higher than the location probability of 0.35 for Grande Comore.

These results therefore suggest that viruses from the SWIO islands have likely not made an appreciable contribution to the diversification of CMG’s on the African continent. Moreover, these results indicate that Grande Comore and Mayotte are the main pathways via which CMG’s are distributed from Africa to the SWIO islands of Mohéli and Anjouan. That Grande Comore and Mayotte are the likely launch pads of CMG movements throughout the Comoros, is probably best explained by these islands being the main traffic hubs in the archipelago and the closest to Africa and Madagascar respectively.

Regarding Madagascar, previous studies reported a severe epidemic of CMD in the 1930s-1940s [37] and the co-existence of ACMV, EACMV and SACMV species [38] based on serological test and partial sequencing. Since no CMG sequences were available from Madagascar at the time of our analysis, we were unable to determine how this island contributes to the dissemination of CMG’s across the SWIO. Obtaining full genome sequences from this island is therefore a top priority in our future efforts to trace CMG movements across Africa and the SWIO islands.

### Complex patterns of component re-assortment

As was abundantly apparent from our phylogenetic and phylogeographic analyses of CMG DNA-A and DNA-B components, the cognate component pairs of particular viruses share neither completely congruent phylogenies nor identical migration histories (Figure 4). We therefore sought to determine whether frequent genome component re-assortment might account for these observations and if so, to what extent had this pseudo-recombination process impacted the evolution of CMG’s. Towards this end we associated within a single dataset couples of DNA-A and B sequences sampled from the same plant. As the co-infections of individual sampled plants with multiple DNA-A and DNA-B components could bias this analysis, we discarded plants from which more than a single genetically uniform DNA-A or DNA-B sequence were isolated, resulting in a dataset with 81 sequence pairs (see Additional file 1: Table S1 for details). It must be stressed, however, that we are unlikely to have discounted all co-infections by this screen and that we remained tentative about our pairing of cognate DNA-A and DNA-B components.

From this dataset, two different analyses were performed: The first made use of the recombination detection algorithms available in RDP3 [39] but with a particular set-up so that only re-assortment events with or without CR exchanges were detected as recombination events. The second analysis approach used the program BEAST’s ancestral state reconstruction capabilities [32] to infer past association histories between species classified DNA-A molecules and DNA-B component sequences. Based on the DNA-A phylogeny, six different DNA-A types were defined (respectively ACMV, EACMV, EACMV-UG, EACMKV, EACMCV and EACMZV) and each of these was assigned as a discrete character state to each of the DNA-B sequences. Therefore, just as with a phylogeographic analysis for which each sampled viruses is associated with a “location state” such as a country, island or province, in our analysis, each DNA-B sequence was associated with one of the six defined “DNA-A states”. We then interpreted individual “migration events” between these six DNA-A states in subsequent phylogeographic analyses of this dataset as individual inter-species re-assortment events.

From the RDP analysis, we detect a total of 15 recombination events that were suggestive of component re-assortment, among which at least four of these events seem to have additionally been associated with the overwriting of a DNA-B CR sequence by that of the DNA-A sequence that newly captured it (Table 2). Most of these full DNA-B re-assortment events (7 out of 11) involve re-associations of DNA-B components between DNA-A sequences of the same species, the three remaining events involving exchanges between EACMV and EACMKV. These results tend to confirm that re-assortment occurs preferentially between closely related viruses, and probably depends on the ability of the DNA-A encoded Rep to trans-replicate the DNA-B sequences with which it comes into contact.

**Table 2:** List of pseudorecombination events inferred in concatenated DNA-A and DNA-B sequences

**Event number**	DNA-B clade *	**Alignment position**		DNA-A *	Original DNA-A *	**B part**^**ϕ**^	**Methods**^**α**^	**P-value**^**β**^
		**Begin**	**End**					
1	EACMV-like	2858	6017	EACMV-UG	EACMV	Full B	RGBMcS**T**	1.9x10^-71^
2	EACMV-like	2883 ^$^	6017	EACMKV	EACMV	Full B	RG**B**MCST	9.9x10^-24^
3	EACMV-like	2883 ^$^	6017	EACMKV	EACMV	Full B	**R**GMCST	6.7x10^-61^
4	EACMV-like	2883	6017 ^$^	EACMV	EACMV	Full B	RBGMC**S**T	1.4x10^-28^
5	EACMV-like	2883 ^$^	6017	EACMKV	EACMV	Full B	RG**B**MCST	1.7x10^-63^
6	EACMV-like	3635	5950	EACMV-UG	EACMZV	pCR+ORFs	RG**B**MCST	2.7x10^-38^
7	EACMV-like	2883 ^$^	5973 ^$^	EACMV	EACMV-UG	Full B	R**G**BMCST	2.2x10^-36^
8	EACMV-like	2883 ^$^	6017	EACMV-UG	EACMKV	Full B	RG**B**MCST	8.1x10^-51^
9	EACMV-like	2820	5924	EACMV	EACMV	Full B	GBMC**S**T	7.3x10^-26^
10	EACMV-like	2883	6017	EACMV	EACMV	Full B	RGbMCS**T**	2.9x10^-12^
11	EACMV-like	2883	6017	EACMV	EACMV	Full B	G**M**CST	8.7x10^-8^
12	EACMV-like	3711	6017	EACMZV	EACMZV	pCR+ORFs	GMC**S**T	8.0x10^-25^
13	EACMV-like	3689	6017 ^$^	EACMV	EACMZV	pCR+ORFs	RGBMc**S**T	8.6x10^-13^
14	EACMV-like	3789	5501 ^$^	EACMKV	EACMZV	pCR+pORFs	RB**M**CST	2.1x10^-6^
15	EACMV-like	1	2201 ^$^	EACMV	EACMV	Full B	rbMC**S**T	1.3x10^-4^

^*^ EACMV-like comprises B components associated with EACMV, EACMKV, EACMZV and SACMV.

^$^ Breakpoints not inferred by RDP.

^α^ R: RDP; G: GENECONV; B: Bootscan; M: MaxChi; C: Chimaera; S: SiScan; T: 3Seq. Upper-case letters imply that a method detected recombination with a multiple comparison corrected P-value < 0.05, lower-case letters imply that the method detected recombination with a multiple comparison corrected P-value > 0.05

^β^ The reported P-value is for the method in bold type and is the best P-value calculated for the region in question.

^ϕ^ The portion of the DNA-B “transferred” between the DNA-A sequences during each re-assortment event is indicated (p: partial; CR: Common Region; ORFs: Open-Reading Frames).

Interestingly, the four detected events which bear signals of CR rewriting involve EACMZV DNA-B components, which seem to form a sub-clade among the other EACMV-like DNA-B sequences. This suggests that there may exist some incompatibility between EACMZV DNA-B CR sequences and the expressed Rep proteins of other EACMV-like viruses (i.e. non-EACMZV viruses), which, without CR overwriting, could inhibit full component re-assortments involving EACMZV variants.

Also consistent with the hypothesis that component re-assortments are likely to be most permissible between closely related viruses is the fact that no clear signal of re-assortment involving EACMCV DNA-B or ACMV DNA-B components, which are clearly distinct from the other DNA-B components, was detected in our analysis.

**Figure 5:**
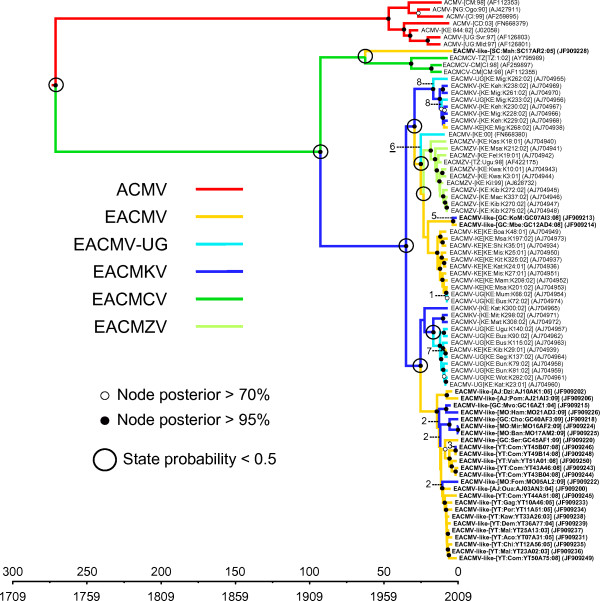
**Analysis of EACMV-like component re-assortments.** Analysis of component re-assortment through counts of DNA-B component “migrations” between the DNA-A sequences of the major CMG lineages. In the analysis DNA-A sequences were classified into six discrete groups. Branches are coloured according to which of these six groups the DNA-B sequence represented by the node to their right was associated with. Black circled nodes indicate ancestral DNA-B sequences where there is less than a < 0.5 state probability for all six of DNA-A lineages (i.e. it is particularly uncertain which DNA-A these ancestral DNA-B sequences were associated with). Whereas filled circles associated with nodes to the right of branches indicate > 95% posterior probability support for these branches, open circles indicate branches with > 70% posterior support. Nodes to the right of branches with < 70% support are left unlabelled. DNA-B “migrations” between different DNA-A lineages that could be explained by genome re-assortment events inferred in an independent RDP3 analysis are indicated by the corresponding RDP3 event number listed in Table 2. The underlined event number (event 6) corresponds to a partial DNA-B”migration” that likely involved overprinting of part of the DNA-B CR with that of the DNA-A sequence that captured it.

Importantly, our second BEAST-based approach provides results completely consistent with our RDP3 based analysis, with the inference of multiple recent (between 1979 and 2009; 95% HPD between 1965 and 2009) DNA-B exchanges between EACMV-UG, EACMV and EACMKV (BFs ranging from 4 to 253; Figure 5). Colour changes over the tree indicate possible inter-species re-assortment events. Importantly, for several nodes (circled in black on Figure 5), probabilities of association of DNA-B’s with particular DNA-A’s were low so that it is impossible to confidently retrace all the association histories. For example, due to its out-group position over the tree, ACMV-like DNA-A is identified as the most probable last common ancestor of all cognate DNA-A sequences. The reason for this is that despite our static designations of the analysed DNA-A sequences to six contemporary lineages, as one progresses from the branch tips into the deeper recesses of the tree, so the meaning of the static contemporary lineage designators becomes less and less meaningful – i.e. ideally the lineages should dynamically change to reflect the fact that, like the DNA-B lineages described in the tree, the DNA-A lineages upon which the designations are based also coalesce as one travels backwards in time down the tree. Despite this unavoidable departure from reality, among the thirteen well-supported re-assortment events detected by this analysis, ten were also supported by the RDP-based re-assortment analysis.

Probably because of the high degrees of similarity between EACMV and EACMKV DNA-B sequences, the analysis revealed reciprocal DNA-B movements between these species on the SWIO islands, comparable to those observed on the continent. Despite having a DNA-B that is also similar to those of EACMV and EACMKV, EACMV-UG being absent from these islands, no related DNA-B exchanges involving this species were detected. Nevertheless these results collectively reiterate that re-assortment of DNA-B components amongst the EACMV-like CMG’s is likely facilitated by these viruses having broadly compatible DNA-A and DNA-B components.

## Conclusions

Here we have analysed the diversity of CMG’s on the Comoros and Seychelles archipelagos, retraced the movement dynamics of these from mainland Africa across these island chains, and, along the way, identified numerous instances of genome component re-assortment. Although the short duration over which the analysed sequences were sampled meant it was not possible to accurately date when migration events had occurred, it was apparent that all three of the EACMV-like species isolated from the SWIO islands (EACMV, EACMKV and EACMCV) were probably recently introduced there from East Africa: a site that is becoming widely recognised as the centre of CMG diversity [12]. Crucially within the Comoros island chain it was very clear that the main islands in this archipelago, Grande Comore and Mayotte, are both the primary conduits through which viruses from Africa gain access to the Comoros, and the main starting points of movements throughout the archipelago.

Although we detected no homologous recombination events between indigenous island viruses and the African CMG’s recently transported to these islands, we did detect ample evidence of both recombination amongst and genome component re-assortments between the island and mainland African CMG’s. These results suggest that besides the epidemiological interactions amongst viruses in the CMG complex, there most likely also exists a strong cohesive evolutionary or ecological interaction between the various CMG species.

Our study has also spawned some interesting questions about the temporal component of the CMG movement dynamics that we have observed. The identification of EACMV-like virus lineages in the Comoros and Seychelles archipelagos that could conceivably have only been transmitted to these islands as recently as 20 to 30 years ago fits well with the upsurgent CMG epidemics in East Africa at approximately this time, that were likely spawned at least in part by a combination of recombinant/re-assortant CMG’s and population explosions of CMG transmitting cassava adapted whiteflies [1]. Whether or not these well-studied events on mainland Africa were mirrored on the SWIO islands but went undocumented remains uncertain since we cannot be entirely confident in the utility of our dataset for accurately dating even the events of 30 years ago. It is plausible that either CMG datasets sampled over greater periods (perhaps exploiting herbarium samples from the 1950s or 60s), or the discovery of extant descendants of distinct “pre invasion” SWIO CMG species through more thorough sampling of all the SWIO islands (including remoter islands such as Madagascar, Mauritius and Reunion) could cast more light on this most interesting era in CMG evolution.

Finally it is interesting to note that the major (or at least the most obvious) routes of viral movement between the SWIO islands also happen to be the main routes of human movement and commercial trade. For now, determining the relative contributions of human *vs.* insect mediated short and long-range CMG movements within and between the SWIO islands, remains just out of reach. It is, however, entirely likely that the future application of slightly more sophisticated phylogeographic models to moderately bigger CMG datasets that include full genome sequences sampled over 40 or 50 years will enable us to very precisely contextualise the relative contributions of humans and whitefly mediated CMG movements during the past 50 years.

## Methods

### Sampling, cloning and sequencing of partial and full CMG genomes from Comoros and Seychelles

Samples of cassava leaves displaying characteristic mosaic symptoms were collected between 2003 and 2009 on the islands Grande Comore, Mohéli, Anjouan, Mayotte in Comoros archipelago and on the islands Praslin, Mahé, and La Digue in the Seychelles archipelago (see Additional file 1: Table S1 for details). Total plant DNA was extracted from dried leaves using DNeasy plant mini kit (QIAGEN France), according to the manufacturer's instructions.

A total of 43 partial genome sequences (Additional file 1: Table S1) were obtained by PCR amplification and cloning of the CP region using either the VD360/CD1266 [40] or AV494/AC1048 [41] primers from total plant DNA extracts as described in [40]. Additionally, 114 full DNA-A and 41 full DNA-B sequences (see Additional file 1: Table S1 for details) were obtained from total plant DNA extracts using the RCA-RFLP strategy [42]. Full genome amplicons were digested using the restriction enzymes *Bam*HI, *Apa*I, or *Eco*RI before ligation into similarly linearized pGEM-3zf or -7zf vectors (Promega, USA) and transformed into *E. coli*.

The resulting clones were sequenced by Sanger sequencing methods with primer-walking by a commercial company (Macrogen, Europe). Sequences were edited and assembled with DNAMAN (version 5.2.9; Lynnon Corporation, Canada) and DNA Baser software (Heracle BioSoft S.R.L., Romania).

### Phylogenetic analyses

Alignments of the 114 DNA-A and 41 DNA-B sequences along with, respectively, 190 and 97 representative begomovirus DNA-A and DNA-B sequences available from GenBank were performed using the Muscle alignment tool available in Geneious (Biomatters Ltd, New Zealand) and edited by eye. In order to assign sequences to known virus species and strains, a pairwise distant matrix was first computed using Geneious with pairwise deletion of gaps. Maximum Likelihood trees were constructed using PHYML 3.0 [43] with the GTR + G_4_ model (selected as the best-fit model using RDP3) and Shimodiara-Hasegawa approximate likelihood ratio tests (SH-aLRT) statistics to assess the robustness of branches. Trees were edited in FigTree v1.3 (available from http://beast.bio.ed.ac.uk/FigTree).

### Recombination and genome component re-assortment analyses

Detection of potential recombinant sequences, identification of likely parental sequences and localisation of recombination breakpoints were carried out using RDP [44], GENECONV [19], BOOTSCAN [45], MAXIMUM CHI SQUARE [46], CHIMAERA [47], SISCAN [48], and 3SEQ [49] recombination detection methods implemented in RDP 3 [39].

This analysis was performed on two different datasets. The first consisted of our new DNA-A sequences (114 sequences) aligned together with all available African begomovirus DNA-A sequences (171), all curtovirus sequences (17), all legumovirus DNA-A sequences (75) and a topocuvirus sequence (1) in GenBank in July 2010. The second dataset contains our 41 new DNA-B sequences aligned together with all available CMG DNA-B sequences available in GenBank in July 2010 (127 sequences). In order to focus the analysis on recombination events involving CMG sequences, signals of recombination were only sought within sequence triplets where at least two of the sequences were CMG sequences. Default settings for the different detection methods and a Bonferroni corrected P-value cut-off of 0.05 where used. Only recombination signals detectable by three or more of the seven applied recombination detection methods and with associated phylogenetic support were accepted as robust evidence of recombination. The breakpoint positions and recombinant sequences inferred for each potential recombination event were manually checked and adjusted when necessary.

We also used RDP3 to detect genome component re-assortment events. For the analysis of component re-assortment, a dataset of 28 Comoros and Seychelles sequences and 53 CMG database sequences was created by concatenating DNA-A sequences with their cognate DNA-B’s to constitute a set of 81 approximately 5600 bp long sequences. Whereas the DNA-A sequences were arranged within the concatemer linearized at the first nucleotide of the AV1 ORF, the DNA-B sequences, similarly linearized on the first nucleotide of the BV1 ORF were arranged 3’of the DNA-A sequence. In so doing, the CR of each component was maintained as single block facilitating the detection of component re-assortment events that additionally involved the overprinting of the DNA-B CR with that of the capturing DNA-A. The analysis was performed with default RDP3 settings except that a window size of 150nts was chosen for the MAXCHI, CHIMAERA, and RDP methods so as to detect only large “recombination events” corresponding to sequence exchanges of approximately component sized genome fragments. Only events detected with three or more methods were considered as credible evidence of recombination. As above, the breakpoint positions and recombinant sequences inferred for each potential recombination event were manually checked and adjusted when necessary.

### Phylogeographic analyses

The spatial dynamics of EACMV-like viruses were reconstructed using a discrete diffusion model implemented within the Bayesian inference framework of the computer program BEAST v1.5.4 [32].

Datasets were constituted with all available EACMV-like virus sequences for which both GPS coordinates and sampling dates were available in June 2010. After a first stage of alignment using POA v2 [50] and MUSCLE [51], the alignment was refined using the clustal-W subalignment tool available in MEGA 4 [52] and edited by eye.

A DNA-A Full Genome dataset (FG-A) was constituted with 114 sequences from the Comoros and Seychelles archipelagos along with 112 EACMV-like sequences available from GenBank (Additional file 1: Table S1). From the core CP region (position 532–1014 relative to JF909063) of this FG-A dataset along with an additional 34 partial sequences and one partial sequence available from GenBank, another dataset, called CP, was constituted. As the core CP of EACMV-UG and EACMMV has a recombinant origin and is only distantly related to the EACMV-like viruses, these sequences were removed from the analysis, resulting in a dataset of 213 sequences (Additional file 1: Table S1). While the FG-A dataset contained substantial evidence of inter-species recombination (particularly in the sequences encoding the complementary sense genes), the CP dataset was mostly free of detectable recombination and contained absolutely no evidence of inter-species recombination. Therefore, although it contained fewer phylogenetically informative sites, analyses of the CP dataset were expected to be free of the confounding effects that recombination in the FG-A dataset might have on estimates of substitution rates and sequence divergence times. Finally, a third dataset, called FG-B, was assembled with our 41 DNA-B sequences from the SWIO islands along with 51 EACMV-like DNA-B sequences from GenBank.

Each sequence was assigned to one of the seven discrete geographical groups (see Additional file 4: Figure S3) defined using a hierarchical clustering approach based on a geographical pairwise distance matrix. These geographically discrete locations, the sampling dates of each sequence and the sequences themselves constituted the BEAST input.

For each dataset, we dated ancestral sequences and estimated nucleotide substitution rates using the Bayesian relaxed-clock approach implemented in BEAST with the following general methodology. After choosing the best nucleotide substitution model using RDP3, substitution rates and MRCA were estimated with BEAST using combinations of three molecular clock models (strict-clock, uncorrelated exponential relaxed-clock and uncorrelated log-normal relaxed-clock) and various demographic models (constant, expansion, logistic, exponential and Bayesian skyline models). Wherever possible, all runs were continued until convergence of the various model parameters as adjudged using Tracer v.1.5 (available at http://tree.bio.ed.ac.uk/software/tracer) by manual inspection of parameter estimate traces and the achievement of suitable effective sample sizes for these parameter estimates. We identified the best fit clock and demographic models by (1) using a Bayes factor (BF) test [53,54] of the marginal tree likelihoods using Tracer v1.5 and (2) manual inspection of the estimated standard deviation of the uncorrelated log-normal clock parameter (ucld.stdev, a measure of the degree to which nucleotide substitution rates vary between branches which provides an indication of whether the strict-clock should be accepted or rejected).

Having identified the best-fit clock and demographic models, we carried out a suitable number of independent MCMC runs to achieve convergence both with effective sample size estimates that usually exceeded 200 (usually after 100 million generations, visualised with Tracer v1.5). After combination of the runs with LogCombiner and subsampling to 20,000 samples, Maximum clade credibility (MCC) trees were constructed using TreeAnnotator and visualized using FigTree v.1.3. A Bayesian stochastic search variable selection (BSSVS), described in detail in [32], was performed to estimate well-supported rates of migration between locations with the use of Bayes factor (BF) tests, with BF values > 3 considered representative of significant migration rates.

Importantly, in order to estimate biases due to different sampling sizes between locations, the analyses were also carried out as above but with the location states of the sequences randomized using an additional operator in the MCMC procedure. The location state probabilities of the root node determined during these analyses were compared with those determined for the datasets analysed without the location state randomization setting.

We used tools available from http://beast.bio.ed.ac.uk/Google_Earth to produce a graphical animation in key markup language (kml) file format of the spatial-temporal movement dynamics of ancestral EACMV-like sequences. These supplementary files (Additional files 7, 8 and 9) can be viewed using Google Earth (available in http://earth.google.com).

### Reconstruction of ancestral DNA-A and DNA-B associations

We chose to retrace the association between DNA-A and DNA-B components using the same ancestral state reconstruction approach as the one used in our phylogeographic analysis. Starting with an alignment of DNA-B sequences, the discrete state associated with each DNA-B sequence simply corresponded with the clade that its associated DNA-A belonged to: i.e. ACMV, EACMCV, EACMV, EACMV-UG, EACMZV or EACMKV.

Following the same procedure as described above for the phylogeographic analysis, the re-assortment analysis allowed the inference of “migrations” of the DNA-B component from one DNA-A type to another, with each “migration” event corresponding with a single re-assortment event. The same BSSVS procedure as was applied for the phylogeographic analysis was applied for testing the significance of evidence for re-assortment, with Bayes factor tests identifying well-supported component exchanges.

## Abbreviations

ACMV: African cassava mosaic virus; AJ: Anjouan; CMD: Cassava mosaic disease; CMG’s: Cassava mosaic geminiviruses; CP: Partial DNA-A dataset-Capsid Protein ORF; EACMCV: East African cassava mosaic virus; EACMKV: East African cassava mosaic Kenya virus; EACMMV: East African cassava mosaic Malawi virus; EACMZV: East African cassava mosaic Zanzibar virus; EACMV: East African cassava mosaic virus; EACMV-like viruses: EACMV, EACMZV, EACMMV, EACMKV, EACMCV; FG-A: Full Genome DNA-A dataset; FG-B: Full Genome DNA-B dataset; GC: Grande Comore; ICMV: Indian cassava mosaic virus; MO: Mohéli; SACMV: South African cassava mosaic virus; SC: Seychelles; SLCMV: Sri Lankan cassava mosaic virus; YT: Mayotte.

## Competing interests

The authors declare having no competing interests.

## Authors' contributions

Conceived and designed the experiments: PL, BR, A, DPM, JML Collected samples: ALA-K, C. A-C, J.-ML. Performed the experiments: JV, EV, MH, MH. Analyzed the data: ADe B, JV, PL, GH, AV, DPM. Wrote the paper: A De B, JV, PL, GH, AV, DPM, J-ML. All authors read and approved the final manuscript.

## Supplementary Material

Additional file 1 **Table S1.** List of the sequences used in the phylogeographic analyses. For each sequence, accession number, sampling date, source, geographical cluster and datasets including the sequence are informed. Additionally, for sequences used in the pseudo-recombination analysis, associated components are indicated. Click here for file

Additional file 2 **Figure S1.** Maximum Likelihood phylogenetic tree of CMG DNA-A sequences. The robustness of each branch is assessed using approximate likelihood ratio test statistics. Different species are indicated with different colours. The tree is rooted with the Sri Lankan cassava mosaic virus (SLCMV) and Indian cassava mosaic virus (ICMV) group. Click here for file

Additional file 3 **Figure S2.** Maximum Likelihood phylogenetic tree of CMG DNA-B sequences. The robustness of each branch is assessed using approximate likelihood ratio test statistics. The tree is rooted with the SLCMV/ICMV and ACMV groups. Click here for file

Additional file 4 **Figure S3.** Distribution of recombination breakpoints detected within (A) DNA-A and (B) DNA-B sequences. All estimated breakpoint positions are indicated by small vertical lines at the top of the graph. A 200-nucleotide window was moved along the alignment one nucleotide at a time and the number of breakpoints detected within the window region was counted and plotted (solid line). The horizontal lines at the top of each graph indicate 99% and 95% confidence thresholds for globally significant breakpoint clusters. Light and dark grey areas respectively indicate local 99% and 95% breakpoint clustering thresholds, taking into account local regional differences in sequence diversity that influence the ability of different recombination detection methods to identify recombination breakpoints. Red areas indicate recombination hot-spots. Click here for file

Additional file 5 **Figure S4.** Geographical distance based clustering of FG-A, CP and FG-B sequence datasets. Groups are indicated on the figure and were used as discrete geographical locations for phylogeographic reconstructions in BEAST. Click here for file

Additional file 6 **Figure S5.** Maximum clade credibility trees constructed from the EACMV-like DNA-A Full genomes (FG-A) dataset. Branches are coloured according to the most probable location state of their descendant nodes, with black circled nodes indicating a state probability < 0.5. The time-scale of evolutionary changes represented in the tree is indicated by the scale bar below it. Whereas filled circles associated with nodes to the right of branches indicate > 95% posterior probability support for these branches, open circles indicate branches with > 70% posterior support. Nodes to the right of branches with < 70% support are left unlabelled. The bar graph on the left corner indicates the root location probabilities. Grey bars represent the probabilities obtained with randomisation of the tip locations. Probable introduction events from Africa to the SWIO islands are indicated with red arrows. Note the bicoloured branch for which uncertainty is highlighted as it is very uncertain whether the location state is East/Centre Africa or Grande Comore (an important distinction when attempting to infer the number of migrations between Africa and the SWIO islands). Click here for file

Additional File 7 **KML file of CP dataset phylogeography analysis.** Lines between locations represent branches in the MCC tree along which location transition occurs. Location circle diameters are proportional to the number of MCC branches maintaining a particular location state at each time-point. The yellow-orange color gradient informs the location state probability (low-high). Altitude of each line is proportional to the time elapsed between its nodes. KML files can be visualized in Google Earth (http://www.google.com/earth/index.html). Click here for file

Additional File 8 **KML file of FG-A dataset phylogeography analysis.** Lines between locations represent branches in the MCC tree along which location transition occurs. Location circle diameters are proportional to the number of MCC branches maintaining a particular location state at each time-point. The yellow-orange color gradient informs the location state probability (low-high). Altitude of each line is proportional to the time elapsed between its nodes. KML files can be visualized in Google Earth (http://www.google.com/earth/index.html). Click here for file

Additional File 9 **KML file of FG-B dataset phylogeography analysis.** Lines between locations represent branches in the MCC tree along which location transition occurs. Location circle diameters are proportional to the number of MCC branches maintaining a particular location state at each time-point. The yellow-orange color gradient informs the location state probability (low-high). Altitude of each line is proportional to the time elapsed between its nodes. KML files can be visualized in Google Earth (http://www.google.com/earth/index.html). Click here for file
