# Supplementary figures and images for: East African cassava mosaic-like viruses from Africa to Indian ocean islands: molecular diversity, evolutionary history and geographical dissemination of a bipartite begomovirus
Source: BMC Evol Biol. 2012 Nov 27;12:228. doi: 10.1186/1471-2148-12-228 (PMC3560262; doi:10.1186/1471-2148-12-228)

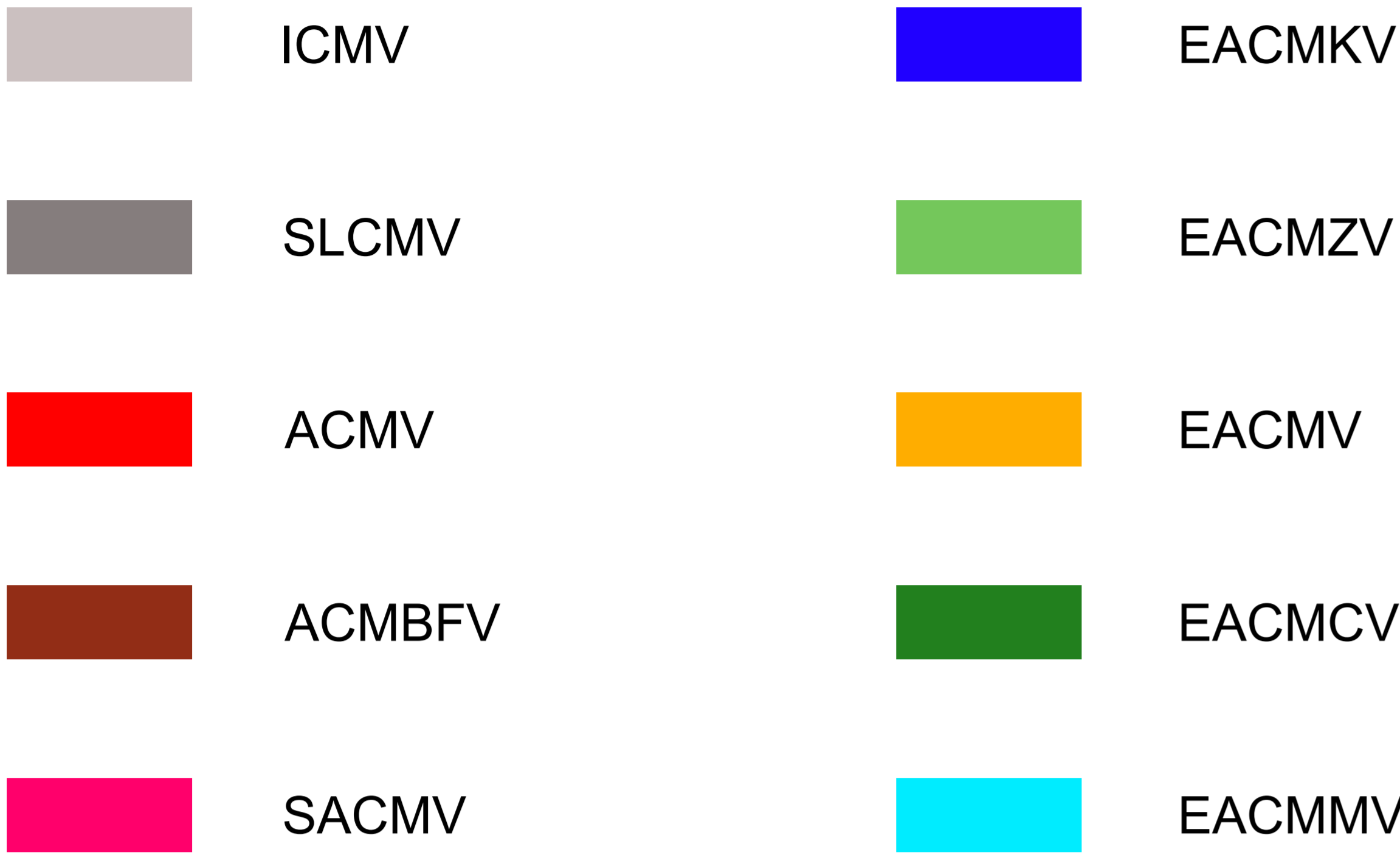

Accession number Sequences from this study

- aLRT > 0.95
- aLRT > 0.70

0.2

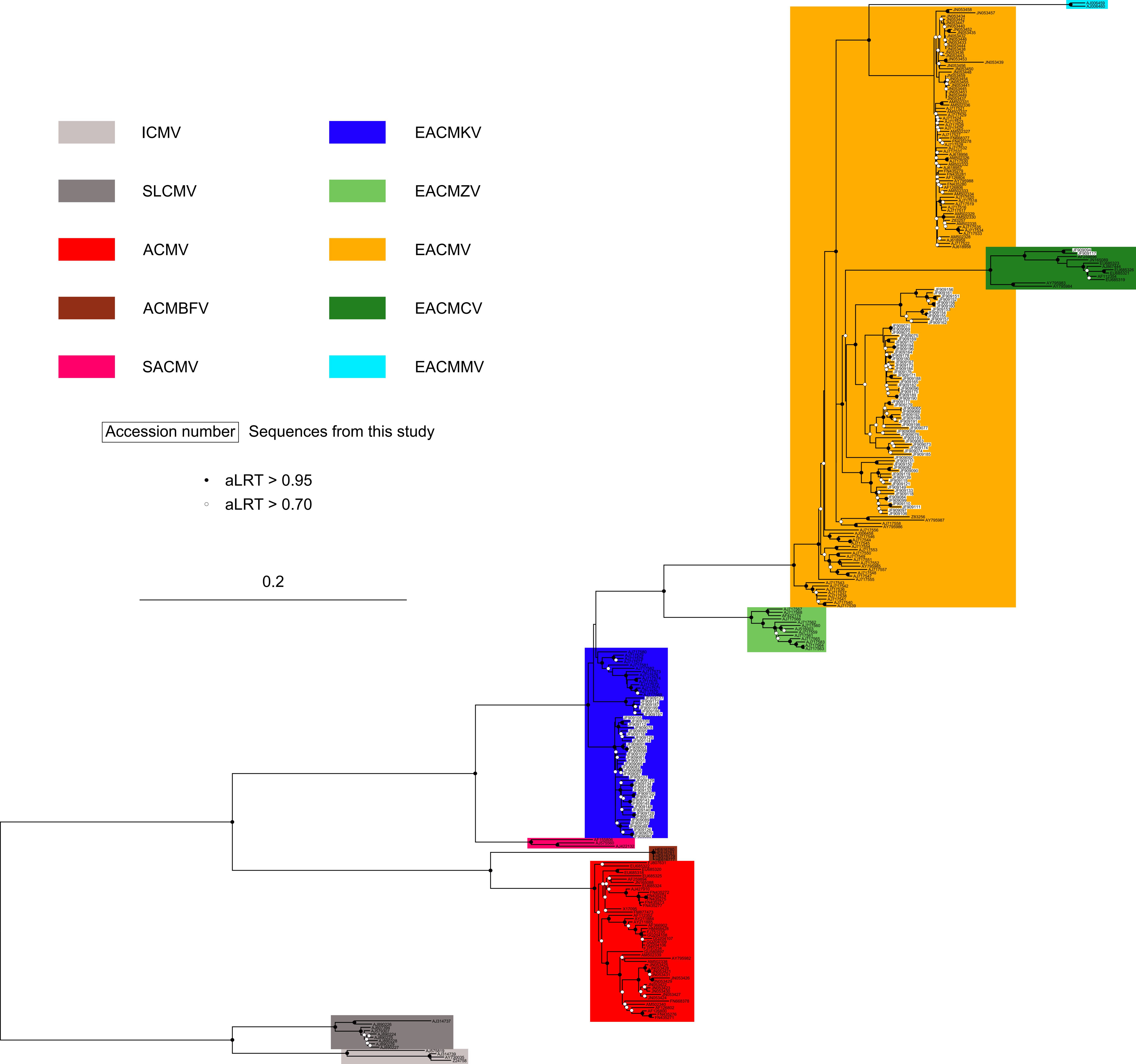

Supplement: Additional file 2 — Figure S1. Maximum Likelihood phylogenetic tree of CMG DNA-A sequences. The robustness of each branch is assessed using approximate likelihood ratio test statistics. Different species are indicated with different colours. The tree is rooted with the Sri Lankan cassava mosaic virus (SLCMV) and Indian cassava mosaic virus (ICMV) group. [file 1471-2148-12-228-S2.pdf]

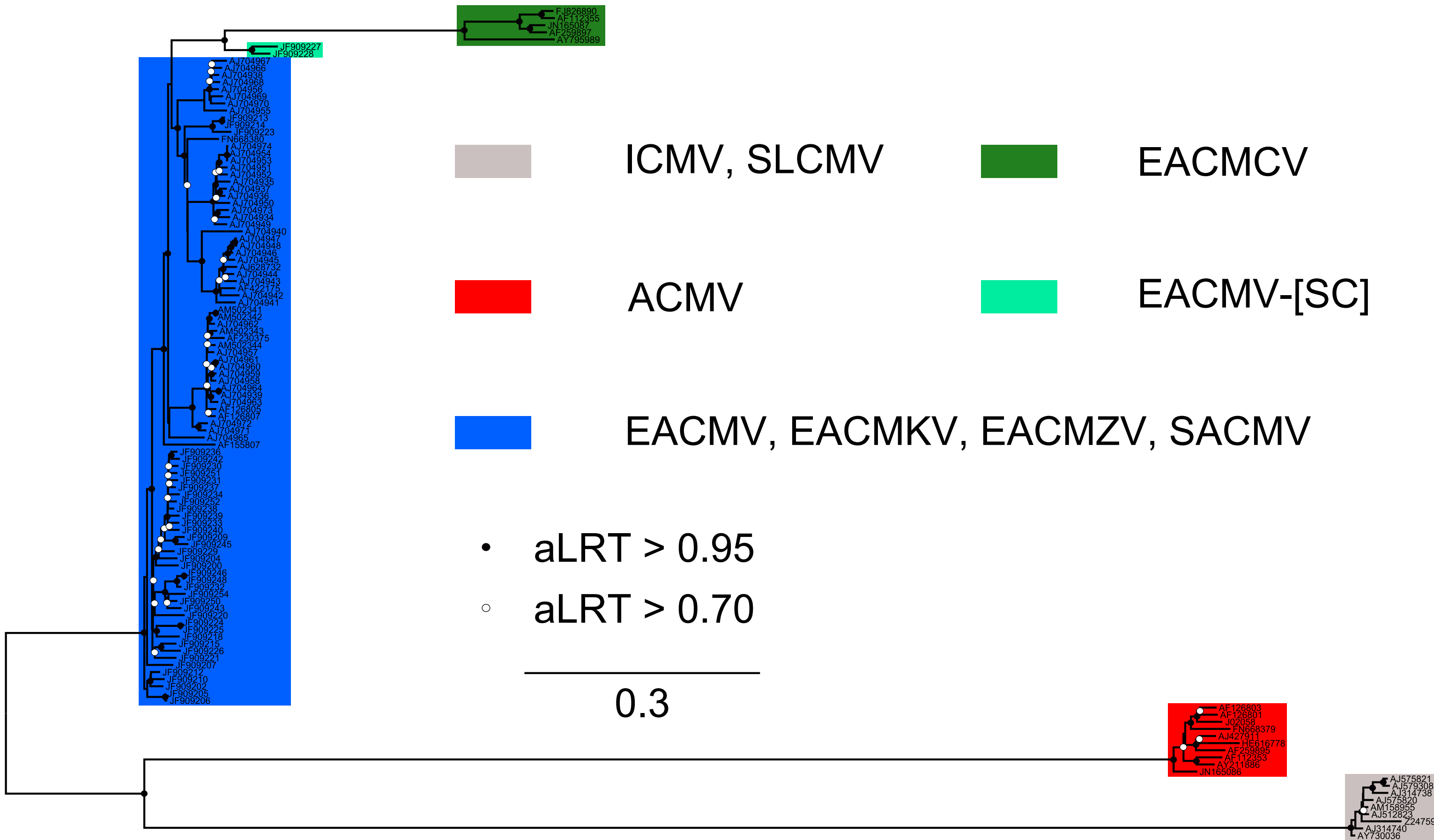

Supplement: Additional file 3 — Figure S2. Maximum Likelihood phylogenetic tree of CMG DNA-B sequences. The robustness of each branch is assessed using approximate likelihood ratio test statistics. The tree is rooted with the SLCMV/ICMV and ACMV groups. [file 1471-2148-12-228-S3.pdf]

**A**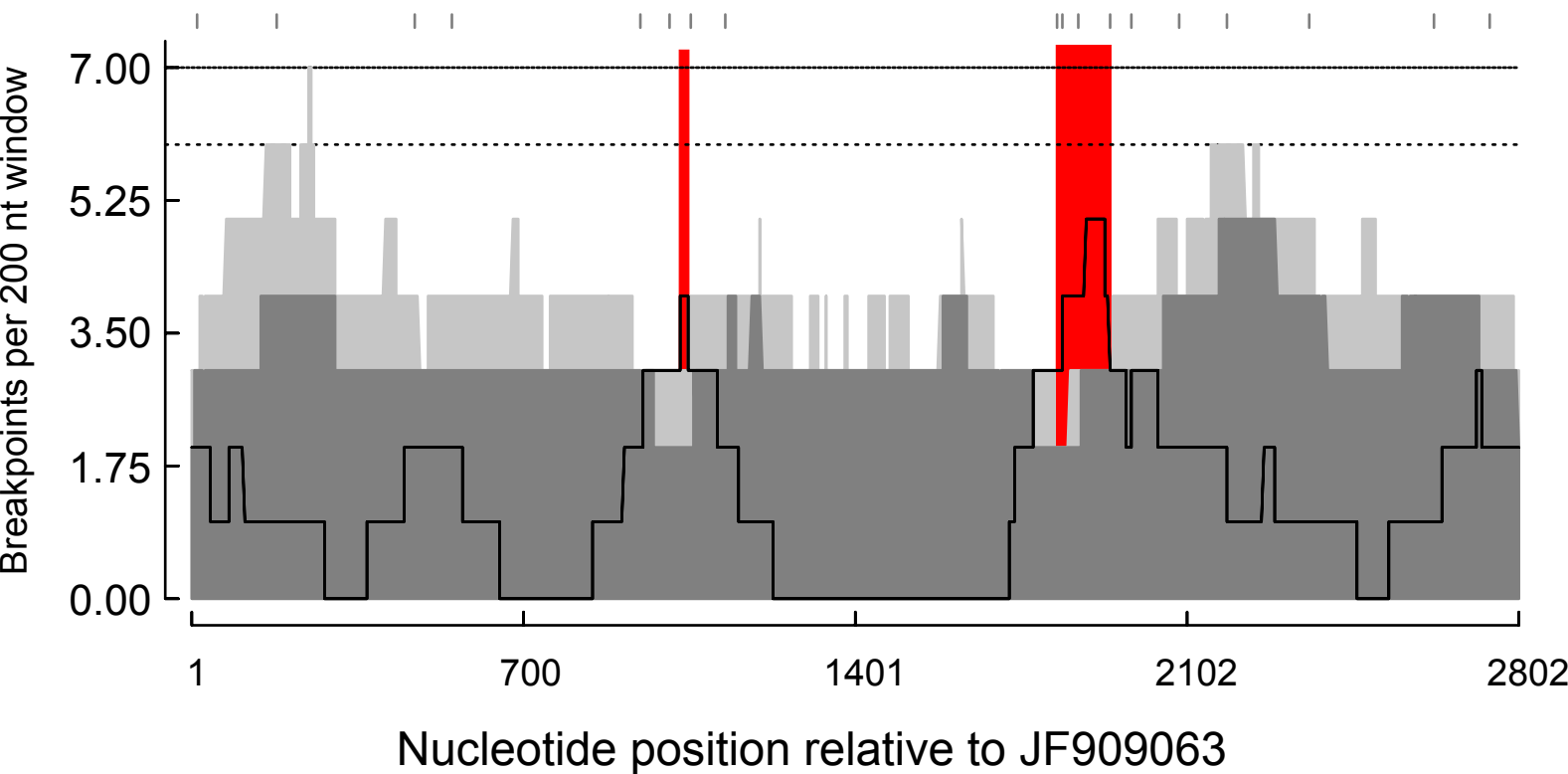**B**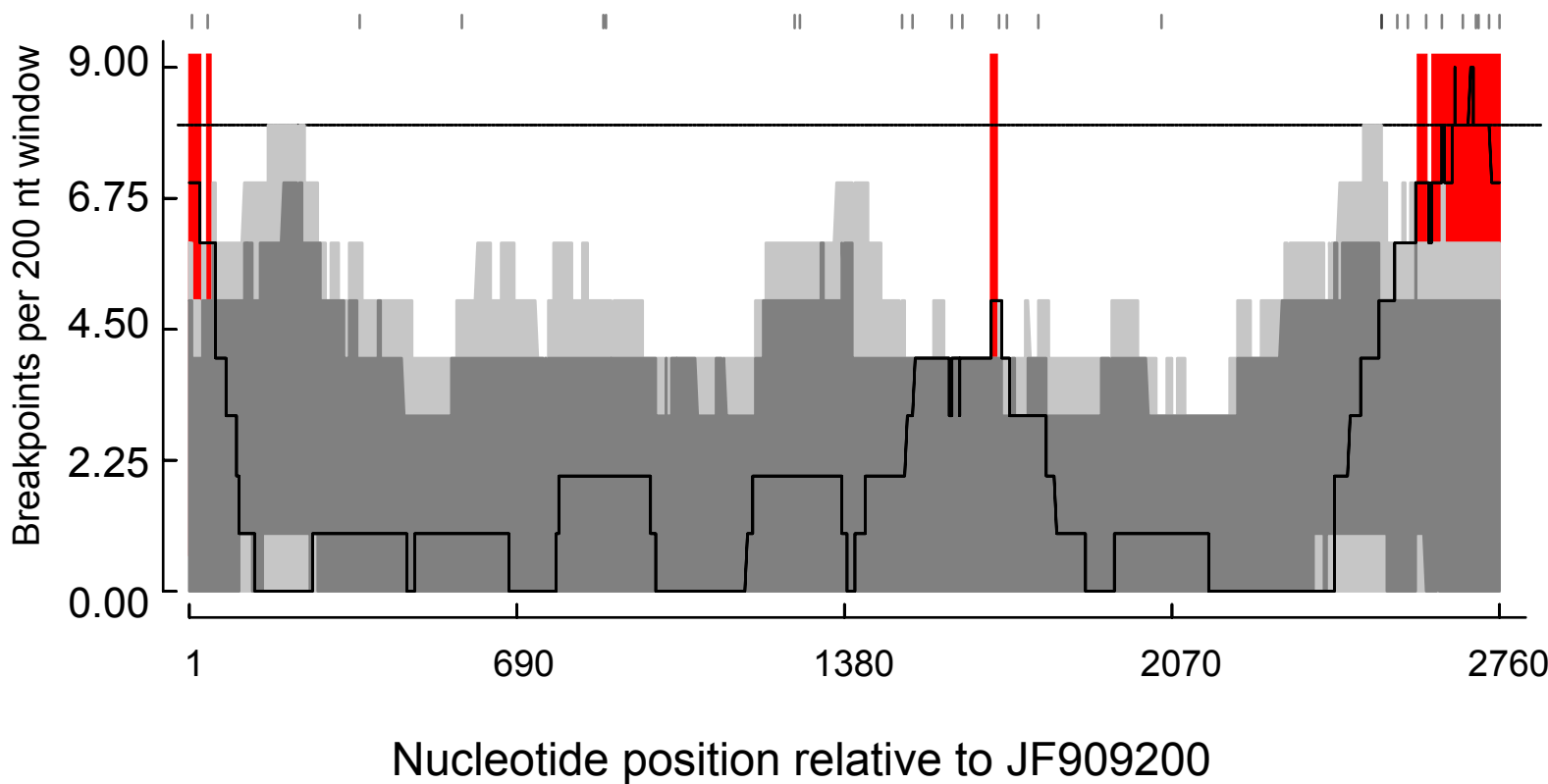

Supplement: Additional file 4 — Figure S3. Distribution of recombination breakpoints detected within (A) DNA-A and (B) DNA-B sequences. All estimated breakpoint positions are indicated by small vertical lines at the top of the graph. A 200-nucleotide window was moved along the alignment one nucleotide at a time and the number of breakpoints detected within the window region was counted and plotted (solid line). The horizontal lines at the top of each graph indicate 99% and 95% confidence thresholds for globally significant breakpoint clusters. Light and dark grey areas respectively indicate local 99% and 95% breakpoint clustering thresholds, taking into account local regional differences in sequence diversity that influence the ability of different recombination detection methods to identify recombination breakpoints. Red areas indicate recombination hot-spots. [file 1471-2148-12-228-S4.pdf]

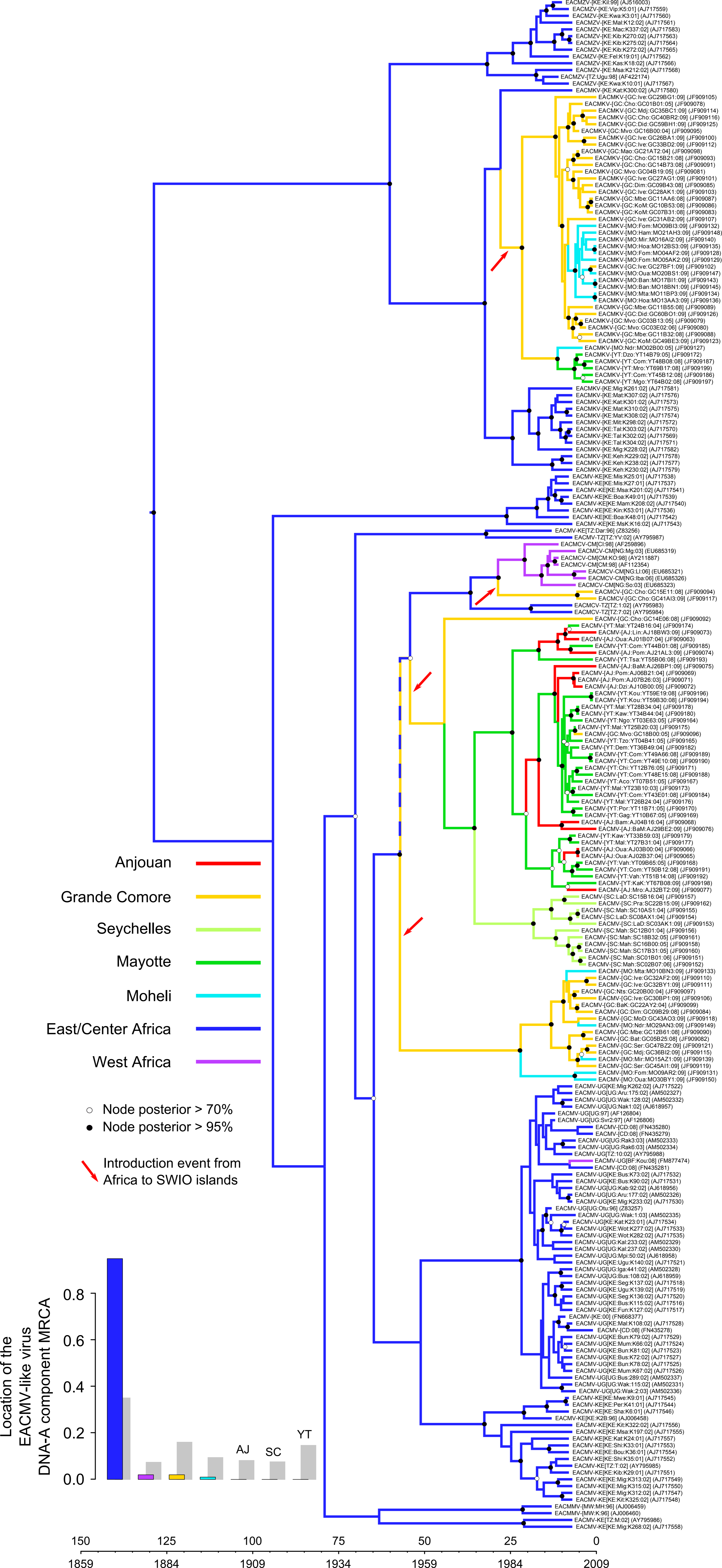

Supplement: Additional file 6 — Figure S5. Maximum clade credibility trees constructed from the EACMV-like DNA-A Full genomes (FG-A) dataset. Branches are coloured according to the most probable location state of their descendant nodes, with black circled nodes indicating a state probability < 0.5. The time-scale of evolutionary changes represented in the tree is indicated by the scale bar below it. Whereas filled circles associated with nodes to the right of branches indicate > 95% posterior probability support for these branches, open circles indicate branches with > 70% posterior support. Nodes to the right of branches with < 70% support are left unlabelled. The bar graph on the left corner indicates the root location probabilities. Grey bars represent the probabilities obtained with randomisation of the tip locations. Probable introduction events from Africa to the SWIO islands are indicated with red arrows. Note the bicoloured branch for which uncertainty is highlighted as it is very uncertain whether the location state is East/Centre Africa or Grande Comore (an important distinction when attempting to infer the number of migrations between Africa and the SWIO islands). [file 1471-2148-12-228-S6.pdf]
