# Supplementary material for: East African cassava mosaic-like viruses from Africa to Indian ocean islands: molecular diversity, evolutionary history and geographical dissemination of a bipartite begomovirus
Source: BMC Evol Biol. 2012 Nov 27;12:228. doi: 10.1186/1471-2148-12-228 (PMC3560262; doi:10.1186/1471-2148-12-228)
Supplement: Additional file 5 — Figure S4. Geographical distance based clustering of FG-A, CP and FG-B sequence datasets. Groups are indicated on the figure and were used as discrete geographical locations for phylogeographic reconstructions in BEAST. [file 1471-2148-12-228-S5.pdf]

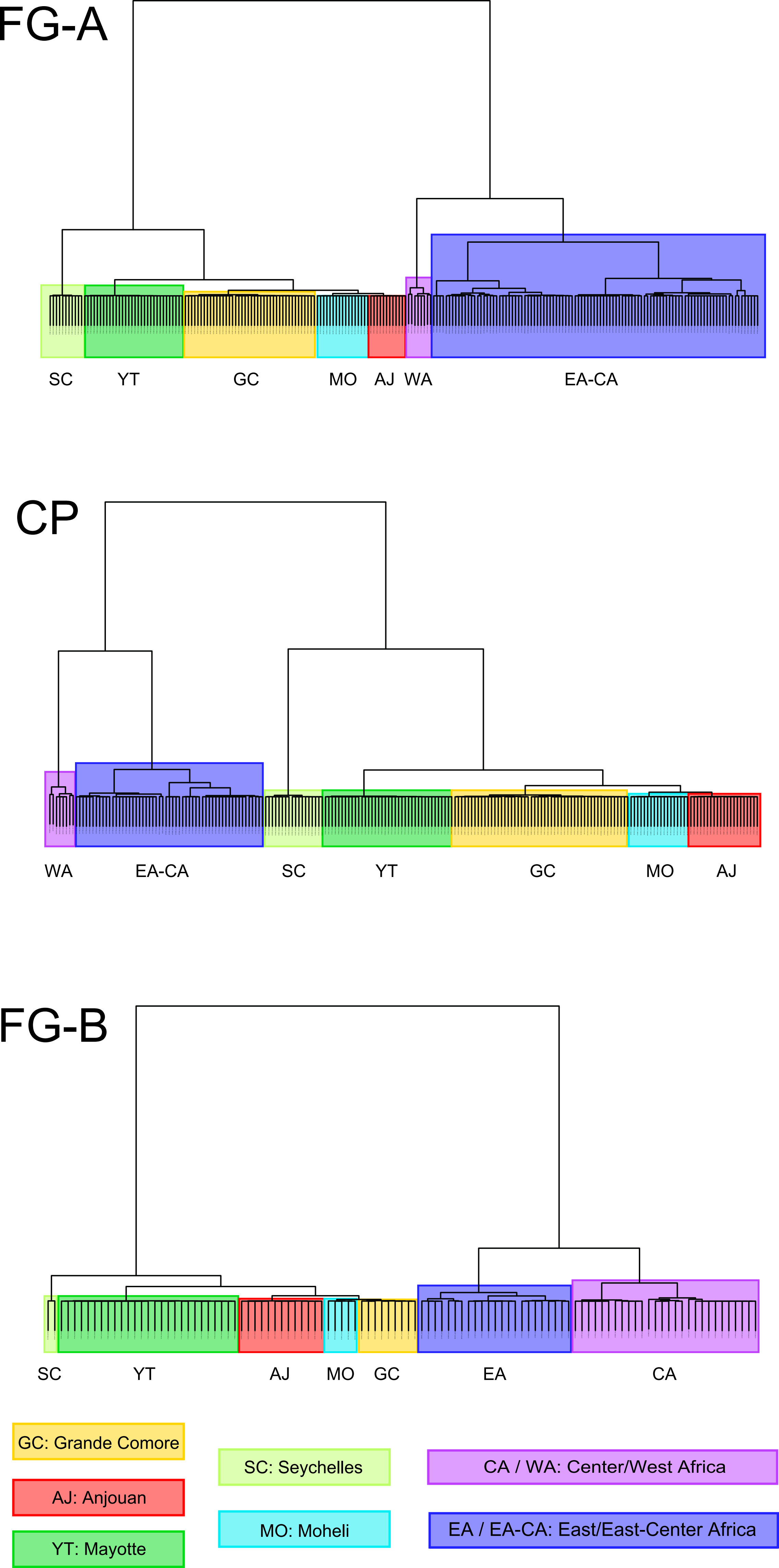

FG-A
SC
YT
GC
MO
AJ
WA
EA-CA
CP
WA
EA-CA
SC
YT
GC
MO
AJ
FG-B
SC
YT
AJ
MO
GC
EA
CA
GC: Grande Comore
AJ: Anjouan
YT: Mayotte
SC: Seychelles
MO: Moheli
CA / WA: Center/West Africa
EA / EA-CA: East/East-Center Africa
